# Incorporation of Dairy Lipids in the Diet Increased Long-Chain Omega-3 Fatty Acids Status in Post-weaning Rats

**DOI:** 10.3389/fnut.2018.00042

**Published:** 2018-05-23

**Authors:** Gaetan Drouin, Daniel Catheline, Annaëlle Sinquin, Charlotte Baudry, Pascale Le Ruyet, Vincent Rioux, Philippe Legrand

**Affiliations:** ^1^Laboratory of Biochemistry and Human Nutrition, Agrocampus Ouest - INRA USC1378, Rennes, France; ^2^Lactalis, R&D, Retiers, France

**Keywords:** dairy lipids, DHA, brain, DPA, infant formula, omega-3

## Abstract

In human nutrition, optimized the status of n-3 long-chain polyunsaturated fatty acids (LCPUFA) and especially docosahexaenoic acid (DHA) during growth appears to be one of the most important goal. We investigated the potential impact of a partial incorporation of dairy lipids (DL) in the diet to increase the n-3 LCPUFA content in tissues, compared to a mixture of vegetable oils. Rats were fed with vegetable oil diet or DL diet, supplemented or not supplemented with DHA, from weaning for 6 weeks. All diets provided the same quantity of 2.3% of total fatty acids of precursor α-linolenic acid. LCPUFA levels in brain, retina, liver, heart, red blood cells and epididymal adipose tissue, Δ-6 desaturase activity and mRNA expression in liver, and plasma cholesterol were measured. Rats fed a DL diet increased their DHA content in brain and retina compared with rats fed a vegetable oil diet and reached the same level than rats directly supplemented with DHA. The status of n-3 docosapentaenoic acid increased with DL diet in heart, red blood cells and liver. The n-3 docosapentaenoic acid specifically discriminated DL diets in the heart. DL diet increased α-linolenic acid content in liver and epididymal adipose tissue, provided specific fatty acids as short- and medium-chain fatty acids and myristic acid, and increased plasma cholesterol. We hypothesized that dairy lipids may increase the n-3 LCPUFA enrichment in tissues by preserving precursor α-linolenic acid from β-mitochondrial oxidation, associated with the presence of short- and medium-chain fatty acids in DL diets. In conclusion, a partial incorporation of dairy lipids in the diet with an adequate α-linolenic acid content improved the n-3 LCPUFA status, especially DHA in brain and retina.

## Introduction

It is well known that n-3 long-chain polyunsaturated fatty acids (LCPUFA) are essential fatty acids (FA) and required for humans throughout life (1–4). Inside the n-3 family, docosahexaenoic acid (DHA) is the most important n-3 LCPUFA in the tissues, especially in the brain and retina (5). Precisely in infants, it has been shown that DHA is essential for brain development and functions (1, 3, 6), as the major component of neural membrane phospholipids (2), and promotes neuroplasticity (7) and learning (8). DHA is also necessary for a good visual development (9, 10) and could prevent cardiovascular diseases (11) and neurodegenerative disorders in adults (12).

The n-3 LCPUFA, as DHA, n-3 docosapentaenoic acid (n-3 DPA) and eicosapentaenoic acid (EPA) can be directly obtained from fish oils and seafood or provided by the conversion from their essential vegetal precursor α-linolenic acid (ALA) (1). The conversion from ALA occurs through a sequence of desaturations and elongations including two enzymes considered as limiting steps: the Δ-6 desaturase and the elongase-2 (13). ALA must also be provided by the diet (14), and the intake of ALA and others n-3 LCPUFA intakes are far lower than the dietary recommendations in Europe (15, 16). Therefore, the conversion rate from ALA to n-3 LCPUFA is not sufficient to provide DHA in adequate amounts in humans (17, 18), as well as in rodents (19). Moreover, several studies showed that DHA content in tissues does not respond linearly to dietary ALA intake (18) and that ALA supplementation results in an increase in EPA and n-3 DPA but not in DHA (20).

The maximal cerebral accumulation of DHA occurs during the first years of life (21). The intake of DHA is provided by breast milk in infants, which contains between 0.2 and 0.4% DHA (22), and which corresponds to the optimum for infant nutrition, as recommended by the WHO for the first 6 months of life. After this period, the milk supply continues, often from commercial infant formulas, whose lipid part is most often composed of a mixture of vegetable oils. More and more infant formulas are supplemented with DHA, as it will be mandatory in a few years in Europe (23). Nevertheless, the direct addition of preformed DHA may be difficult for economic and sustainability reasons, since sources of DHA are still limited (24, 25). For these reasons, it seems crucial to find the optimal nutritional conditions to increase ALA conversion pathway toward derivatives and to the incorporation of DHA in tissues and specifically in the brain and in the retina. The optimization of infant formulas is needed during the period of growth where brain DHA requirements are high.

Some studies showed that infants receiving formulas containing dairy lipids had an intermediate status in n-3 LCPUFA between breastfed infants and infants fed formulas containing a mixture of vegetable oils (26). Moreover, in rodents, the partial incorporation of dairy lipids in diets could improve conversion pathway from ALA to n-3 LCPUFA. However, these studies in rodents were often performed in specific conditions with a deficiency of ALA before the administration of the diet (27, 28), or with different quantities of ALA in diets (7). Dairy lipids also provide numerous species of specific lipids, including cholesterol, and offer a composition closer to breastmilk than a blend of vegetable oils (29).

Providing a higher n-3 LCPUFA amount during growth is essential for optimal development. The purpose of this study was to evaluate the impact of a partial replacement of vegetable oils by dairy lipids, supplemented or not with DHA, on n-3 LCPUFA metabolism in different tissues. This experiment was performed in post-weaning rats fed the same amount of ALA and without any n-3 FA deficiency before the experimental diets. We also investigated the capacity of dairy lipids to provide diversified FA and cholesterol in comparison to vegetable oils.

## Materials and methods

### Chemicals and products

Solvents and chemicals were obtained from Thermo Scientific (Elancourt, France), VWR (Fontenay-sous-Bois, France) or from Sigma (Saint-Quentin-Fallavier, France). Butterfat and oleic sunflower oil were provided by Lactalis group (Retiers, France). High n-3 LCPUFA fish oil (Omegavie® DHA 80 EE Qualitysilver®) was graciously provided by Polaris (Quimper, France).

### Ethyl docosahexaenoate (DHA-EE) purification

The DHA-EE > 99% was obtained from a high n-3 LCPUFA fish oil (Omegavie® DHA 80 EE Qualitysilver®). The purification was performed by liquid chromatography with a puriFlash®4250 system (Interchim, Montluçon, France) with a Chromabond® flash column (RS 330 C_18_ ec, porosity 45 μm, diameter 60 mm, length 200 mm, Macherey Nagel, Hoerdt, France) followed by a Puriflash flash column (PF-15C_18_ HP-F0330, porosity 15 μm, diameter 60 mm, length 226 mm, Interchim). An elution was performed at room temperature in an isocratic mode of methanol/water (95/5, *v/v*) with a constant flow of 120 mL/min. Each fraction of the first injection was collected every 30 s and was evaporated with a vacuum evaporator, then analyzed by gas chromatography-mass spectrometry (GCMS). For the following injections, we pooled fractions which had DHA-EE > 97% purity in the first injection. This fraction was injected under the same conditions in a preparative Interchrom column (Uptisphere strategy C_18_-HQ, porosity 5 μm, diameter 50 mm, length 250 mm, Interchim) with an isocratic mode of methanol/water (98/2, *v/v*). All fractions corresponding to the DHA-EE peak has been collected and pooled to obtain DHA-EE >99% purity. DHA-EE possible peroxidation was controlled performing TBARS assay on DHA-EE oil at the beginning of the experimentation and on diets at the end of the experimentation (30, 31). Malondialdehyde (TBARS) was not detected in DHA-EE oil or in diets.

### Diets

Four different lipid blends (Table 1) were prepared from a combination of commercially available natural oil sources (Leclerc, Rennes, France). Lipid blends were composed of a blend of vegetable oils (VO) or of a blend of 50% vegetable oils and 50% dairy lipids (DL) (*w/w*), not supplemented or supplemented with 0.5% DHA (VO+DHA and DL+DHA). Dairy lipids provided from butterfat. Lipid blends were made with particular attention to ALA and linoleic acid (LA) levels, with a LA/ALA ratio around 5, as nutritionally recommended in humans in France (32). The FA composition of the lipid blends and the oils used in the semi-synthetic diets were designed according to the fatty acid compositions of European infant formulas commercially available. The specific FA present exclusively in dairy lipids, like short- and medium-chain fatty acids (SMCFA), branched-chained and odd FA, were compensated for oleic acid provided by high oleic sunflower oil (Table 1) (33, 34). FA composition of lipid blends was analyzed by GCMS after Folch extraction and derivation as described above in 2.5 (35). The FA composition of VO and DL diets were respectively the same in both experiments.

**Table 1:** Oil mixtures and fatty acid composition of the lipid blends composing the four experimental diets.

**Diets**	**VO**	**VO + DHA**	**DL**	**DL + DHA**
**LIPID SOURCES COMPOSING THE LIPID BLENDS (g/100 g)**				
Butterfat			50	50
Palm oil	35	35		
High oleic sunflower	42	42	22	22
Sunflower oil			7	7
Colza oil	23	23	21	21
DHA-EE^a^		0.5		0.5
**FATTY ACID (FA) COMPOSITION OF THE LIPID BLENDS (%)**				
**Saturated FA**	**21.0**	**20.9**	**37.5**	**37.3**
SMCFA^b^			4.8	4.8
Branched-chain FA^c^			0.9	0.9
C14:0	0.3	0.3	5.5	5.5
C16:0	17.2	17.1	17.1	17.0
C18:0	3.1	3.1	6.7	6.7
Odd FA			0.6	0.6
**Monounsaturated FA**	**65.3**	**65.0**	**48.0**	**47.8**
C16:1 n-7	0.1	0.1	0.9	0.9
C18:1 n-7	1.7	1.7	1.6	1.6
C18:1 n-9	63.3	63.0	43.1	42.9
Others	0.2	0.2	2.4	2.4
**Polyunsaturated FA**	**13.7**	**14.1**	**14.5**	**14.9**
C18:2 n-6 (LA)	11.3	11.2	11.5	11.4
C18:3 n-3 (ALA)	2.3	2.3	2.3	2.3
C22:6 n-3 EE (DHA)		0.5		0.5
Others			0.7	0.7
**LA/ALA ratio**	**5.3**	**5.0**	**5.0**	**5.0**

*All diets contained for 1 kg of diet: 100.0 g of fat (21% of total energy), 220.0 g of HCl-casein, 1.5 g of DL-methionine, 402.3 g of cornstarch, 201.2 g of sucrose, 20.0 g of cellulose, 45.0 g of mineral mix 102 and 10.0 g of vitamin mix 102*.

a *Ethyl esters purified by preparative liquid chromatography*.

b *Short- and medium-chain fatty acids (C4:0 to C10:0)*.

c *iso-C16:0, iso- and ante-C17:0*.

All the diets contained 10% (w/w, 21% of total energy) of lipid blend and 90% (w/w) of a lipid-free base. The lipid-free base was prepared at the Unité de Production d'Aliments Expérimentaux (UPAE, INRA, Jouy-en-Josas, France). The lipid-free base contained: 220.0 g of HCl-casein, 1.5 g of DL-methionine, 402.3 g of cornstarch, 201.2 g of sucrose, 20.0 g of cellulose, 45.0 g of mineral mix 102 and 10.0 g of vitamin mix 102 for 1 kg of diet. Then, 100.0 g of fat/kg of diet of the lipid blend were added in our laboratory.

To prepare the rat foods, the lipid-free base and the lipid blend were mixed then sifted twice, 13% water was added (*w/w*) then the mixture was homogenized and shaped as croquettes. The rat foods were sealed in an airtight package and frozen at −20°C because they were prepared at the beginning of the study. During the experiments, rat foods were thawed at +4°C 24 h before being given to animals. Malondialdehyde concentration was measured in the diets to detect a potential peroxidation of DHA as described above in section Ethyl Docosahexaenoate (DHA-EE) Purification. Malondialdehyde was not detected in diets, neither at the beginning nor at the end of the study. The diets were isoenergetic and isolipidic. Total sterols were measured in DL and VL blends using the Liebermann in order to quantify the cholesterol intake of animals as described previously (36).

### Animals

**Figure 1:**
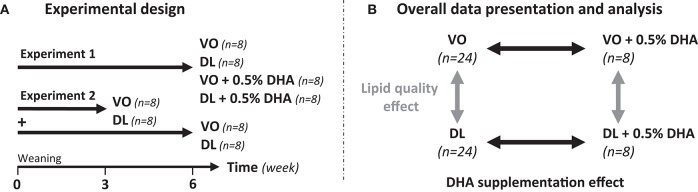
**(A)** Study design including two experiments. In experiment 1 (*n* = 8/group), rats were fed from weaning for 6 weeks with the vegetable oil blend diet (VO) or the vegetable oil and dairy lipid blend diet (DL), not supplemented or supplemented with 0.5% DHA (VO+DHA and DL+DHA). In experiment 2 (*n* = 8/group), rats were fed the VO or the DL diet from weaning for 3 or 6 weeks. The two experiments were performed at 1-year interval. **(B)** Data presentation and statistical analysis strategies. Results are presented as mean ± SEM of combined groups from experiments 1 and 2. Consequently, a linear mixed-model with 2 fixed factors and 2 hierarchized random effects (experimentation/duration of diet) was used for statistical analysis.

This study is composed of two experiments performed at 1-year interval (Figure 1A). In the experiment 1, 32 rats (*n* = 8/group) were fed VO or DL diets not supplemented, or supplemented with 0.5% DHA (VO+DHA and DL+DHA) from weaning for 6 weeks. In the experiment 2, 32 rats (*n* = 8/group) were fed VO or DL diets, not supplemented with DHA from weaning for 3 or 6 weeks. After doing a power calculation based on the brain DHA content difference between the VO and DL groups of the experiment 1, the experiment 2 was realized in order to increase the number of individuals included in the VO and DL groups to increase the power of the study. For both experiments, Sprague Dawley rats (mean body weight 60 ± 1 g, 3 weeks old at the beginning of the experiment) were randomly assigned into the 4 groups and were housed 4 animals by cage on a 12 h light-dark cycle and maintained at 21°C ± 2°C with free access to water and food. Body weight, food and water intakes were measured 3 times a week.

At the end of the experiment, fasted rats were anesthetized with an intraperitoneal injection of sodic pentobarbital (140 mg/kg of body weight) (Euthasol Vet, France). Blood samples were collected by cardiac punctures into heparin-treated vacutainers (Dominique Dutscher, Brumath, France) and plasma was separated from blood cells by centrifugation (3,000 g, 20 min, amb T°) and stored at −80°C. After removing the peripherical blood mononucleated cells layer, red blood cells (RBC) were mixed, weighed and stored at +4°C (37). Whole eyes were removed and stored in 4% formol-PBS at 4°C during 1 week before the dissection of the retina. Tissues were removed, washed with PBS, weighted and separated in two parts: around 200 mg of the left lobe of liver, left ventricle of heart and part of the brain from frontal cortex to striatum were stored at −20°C in 4 mL of dimethoxymethane/methanol (4:1, *v/v*); 50 mg of liver was homogenized with an Ultra-Turrax in RNA-extraction mixture and stored at −80°C; the rest of the organs were snap-frozen in liquid nitrogen and stored at −80°C (38, 39).

All experiments were performed in accordance with the European Union Guidelines for Animal Care and Use (2003/35/CEE). The experimental procedures (n° APAFIS#1389-2015080411586889 v4 and n°APAFIS#5139-201605121200996 v1) were approved by the French Animal Care Committee (Rennes, approval number A3523838) and the Ministry of Higher Education and Research and Innovation, in compliance with recommendations of the 2013–118 directive for animal experimentation.

### Lipid extraction and fatty acid composition

Lipids were extracted twice from tissues with 20 volumes dimethoxymethane: methanol (4:1, *v/v*) after homogenization with an Ultra-Turrax (40, 41). RBC lipids membranes were extracted twice with hexane/isopropanol (3/2, *v/v*) after acidification with HCl 3M, as previously described (42). Brain phospholipids were separated from total lipids on preparative thin-layer chromatography (TLC) using silica gel H (Merck Millipore, Darmstadt, Germany) plates impregnated with a mixture of hexane/diethyl ether/acetic acid (85/15/1, *v/v/v*). Total lipids and phospholipids were saponified by 1 mL of 0.5 M NaOH in methanol at 70°C for 30 min and then methylated with 1 mL of BF_3_ (14% in methanol) at 70°C for 30 min. Fatty acid methyl esters were extracted twice with pentane, washed twice with NaCl 0.9% and were taken up in hexane. Fatty acid methyl esters from brain phospholipids were purified by another step of TLC with a mixture of hexane/diethyl ether (80:20, *v/v*), extracted a first time by a mixture of methanol/hexane/NaCl 0.9% (3:4:3, *v/v/v*), then extracted a second time with 4 volumes of hexane and washed with NaCl 0.9% (43).

GCMS analysis was performed using an Agilent Technologies 7890A (Bios Analytic, Santa Clara, CA, RRID:SCR_015742) with a bonded silica capillary column (BPX 70, 60 m × 0.25 mm; SGE, Melbourne, Australia) containing a polar stationary phase of 70% cyanopropyl polysilphenylene-siloxane (0.25 μm film thickness). Helium was used as carrier gas (average velocity 36 cm/s). The column temperature program started at 150°C and gradually increased at 4°C/min to 250°C, and held at 250°C for 10 min. Mass spectra were recorded with an Agilent Technologies 5975C inert MSD with triple axis detector. The mass spectrometer was operated under electron impact ionization conditions (electron energy 70 eV, source temperature 230°C). Data were obtained in the full scan mode with a mass range of m/z 50–550 atomic mass units (amu), using the MSD Chemstation software E.02.02.14.31 (RRID:SCR_015742). Identification of the FA methyl esters was based upon retention times obtained for a homemade pool of FA methyl ester commercial standards with a purity >99% (Sigma) when available. The National Institute of Standards and Technology database (version 2.0, RRID:SCR_006440) was also sometimes used to identify unknown FA. All identified FA with a signal/noise> 10 were considered in the analysis of the proportions, FA identified with a signal/noise between 3 and 10 were marked as a trace.

### Biochemical parameters of the plasma

Total cholesterol (cholesterol CHOL, Randox Laboratories, Crumlin, UK, RRID:SCR_005525) and high-density lipoprotein cholesterol (HDL-cholesterol) (HDL-CHOL, Randox Laboratories) were assayed with commercial enzymatic kits according to the manufacturer's instructions on heparinized plasma in triplicate. HDL cholesterol levels were measured after precipitation of apolipoprotein-B containing lipoproteins using phosphotungstic acid. Non-HDL cholesterol was calculated by the difference between total cholesterol and HDL-cholesterol.

### Δ-6 desaturase activity assay

The liver was homogenized in a phosphate buffer containing 0.25 M sucrose to recover the post-mitochondrial supernatant, as previously described (36).

Enzymatic activity was determined using a 1-mL assay mixture containing 100 μL of supernatant (100 μg proteins), 150 mmol/L phosphate buffer (pH 7.16), 6 mmol/L NADH, 6 mmol/L MgCl_2_, 7.2 mmol/L ATP and 0.54 mmol/L CoA. The incubation was carried out in duplicate at 37°C for 20 min, after addition of 60 nmol of [1-^14^C]-ALA (60 μmol/L, 10 mCi/mmol, ARC, Saint Louis, MO, USA). The reactions were stopped by adding 1 mL of 2 M KOH in ethanol. After 30 min at 70°C, the FA were liberated by acidification, extracted with diethyl ether, converted to FA naphthacyl esters and separated on HPLC (Alliance 2695 integrated system, Waters, Saint-Quentin-en-Yvelines, France) as previously described (44). Peaks corresponding to radiolabeled FA substrate and product of each desaturase assay were collected and subjected to liquid scintillation counting (PerkinElmer 2810, Waltham, MA, USA). The enzyme activity was determined and expressed as pmol of substrate converted to product per min per mg of protein. Protein in the supernatant used for the desaturase assays was determined by Lowry method (45).

### RNA isolation and real-time qPCR

Total RNA were isolated from 50 mg of the liver with Nucelospin® RNA commercial kit (Machery Nagel) according to the manufacturer's instructions. RNA purity and concentration were verified by colorimetric A260/A280 ratio and A260/A230 ratio with a nano-spectrophotometer (DS11 spectrophotometer, DeNovix, Wilmington, DE, USA), all calculated ratio exceeded 2.0 and 2.2, respectively. Total RNA were retrotranscribed by using the High-capacity cDNA Reverse Transcription kit (Applied Biosystem, Fisher, Illkirch, France) according to the manufacturer's instructions.

Transcript of fatty acid desaturase 2 gene (*Fads2*) coding for the Δ-6 desaturase enzyme was quantified by Real-time qPCR in optical 96-well plates on a CFX 96 real-time PCR Detection System (Biorad, Marnes-la-Coquette, France) with the TaqMan 2X Universal PCR MasterMix (Applied Biosystem) containing 50 ng of cDNA, 500 nM of each forward and reverse primers and 10 μL of MasterMix. Primers and 5′-FAM/TAMRA-3′ Taqman probes specific to *Fads2* gene (forward primer 5′-TCCACAAGGACCCCGACATA-3′; reverse primer 5′- TTGTAGGGCAGATATTTCAGCTTCTT-3′) and TaqMan probe (5′FAM-TTGGAGAGTGGCAGCCCCTCGAGTA-TAMRA3′) were designed (Eurogentec, Angers, France). Amplification was performed over 40 cycles of 95°C for 15 s and 60°C for 1 min. The *18S* expression was quantified as an endogenous control using the 18S RNA Control kit Yakima-Yellow Eclipse Dark Quencher (Eurogentec) and relative gene expression was determined from the threshold cycle (Ct) using the ΔΔCt method (46).

### Statistical analysis

We combined the results of experiments 1 and 2 in order to present and analyze the data (Figure 1B). FA composition of tissues, plasma assays, and organs weight were statistically analyzed with a linear mixed-model with 2 fixed factors (lipid quality, DHA supplementation) and 2 hierarchized random effects (experimentation/duration of diet). The major aim was to compare the potential effect between lipid quality effect (VO and VO+DHA vs. DL and DL+DHA) and DHA supplementation effect (VO and DL vs. VO+DHA and DL+DHA) on tissues FA composition. Consequently, the diet duration effect was included as a random effect, as no interaction was found between lipid quality effect and diet duration effect. Body weight and food consumption were analyzed with the same linear mixed-model for repeated measures. Multiple comparisons between groups were performed by Tukey-Kramer procedure adjusted with false discovery rate method (fdr). Prior to analysis, the model was assessed by verifying the normality of the model and the normality of the residuals graphically, homoscedasticity was supposed true. When normality of model was not validated, groups were compared by non-parametric ANOVA using Kruskal-Wallis test followed with Dunn's *post-hoc* test (PCR and enzymatic activity results). For all procedures, *p* < 0.05 was considered as significant. All data were reported as mean ± SEM.

To identify fatty acid biomarkers across groups, multivariate analysis was used in this study on FA composition of rats fed for 6 weeks. First, principal component analysis (PCA) was performed to study the model overview. Secondly, redundancy analysis (RDA), a variant of partial least square analysis (PLS-DA), was used (47). RDA was performed to assess differences in FA metabolic profiles, considering as constraints the lipid quality effect, the DHA supplementation effect and the interaction between these two factors. The analysis was assessed separately for each tissue. As FA compositions were presented as the percentage of total detected FA, and that the FA proportion took place in a large range of values (0.10–56.74), the data were transformed by centering log-ratio (*clr*), then by centering-scaling (48). A permutation test was used to consider the significance of these three terms (49). This test was followed by pairwise comparisons using factor fitting to an ordination adjusted with fdr method to assess the significance of the 4 diets. FA explaining most of the variability between lipid quality effect (LQ, DL and DL+DHA vs. VO and VO+DHA) or DHA supplementation effect (S, VO and DL vs. VO+DHA and DL+DHA) were chosen by considering the test of Pearson correlation coefficient between FA data and individual scores, and a Pearson correlation coefficient *R*^2^ > 0.5 (50). FA were discussed using the directions in the two-dimensional vector spaces which discriminate these effects (LQ and S), as previously described (27).

Statistical analysis were realized using R software v3.4.2 (RRID:SCR_001905) and R packages *nlme* v3.1.131 (RRID:SCR_015655), *RVAideMemoire* v0.9.68 (RRID:SCR_015657), *chemometrics* (v0.1) and *vegan* v2.4.4 (RRID:SCR_011950).

## Results

### Body weight and food intake

At the end of the experimentation, body weight gains and organ weights did not differ significantly between groups, except for brain weight which was higher in rats fed the diet partially enriched with dairy lipids (DL) in comparison to rats fed the vegetable oils blend diet (VO) (+11% *w/w, p* < 0.01) (see Supplementary Material 1, Table 1). Means of cumulated food, water consumption, and organ weight did not differ amongst the groups (see Supplementary Material 1, Figure 1).

### Dairy lipids differently increased n-3 long-chain polyunsaturated fatty acids (LCPUFA) in tissues

The main objective of this study was to address the impact of DL diets on n-3 LCPUFA content in tissues, compared to VO diets in post-weaning rats fed the same amount of precursors α-linolenic acid (ALA) and linoleic acid (LA). Our study focused on six tissues: brain, retina, heart, liver, red blood cells (RBC) and epididymal adipose tissue (EAT). The complete fatty acids composition of tissues are presented in Supplementary Material 2, Tables 1–5.

**Figure 2:**
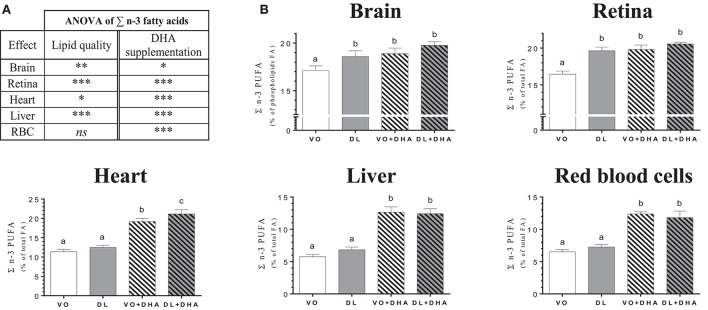
**(A)** Overall lipid quality (LQ) effect and DHA supplementation (S) effect of the sum of n-3 PUFA (ALA+EPA+DPA+DHA) proportions in brain, retina, heart, liver and red blood cells (RBC) of rats fed the vegetable oil blend diet (VO) or the vegetable oil and dairy lipid blend diet (DL), not supplemented or supplemented with 0.5% DHA (VO+DHA and DL+DHA). The significance of the two effects was assessed by two-ways ANOVA (^*^p < 0.05, ^**^p < 0.01, ^***^p < 0.001). ^*^Represents globally an increase in favor of the DL groups for the LQ effect and in favor of the lots supplemented with DHA for the S effect. *ns*, not significant. **(B)** Proportions of the sum of n-3 PUFA (ALA+EPA+DPA+DHA) in tissues. Values are mean ± SEM. Different superscript letters indicate significant differences for multiple comparisons (*p* < 0.05).

Concerning the overall lipid quality and DHA supplementation effects on total n-3 PUFA tissue contents as the sum of n-3 LCPUFA (EPA+n-3 DPA+DHA) and n-3 LCPUFA precursor (ALA) (Figure 2), rats fed DL diets had higher total n-3 PUFA contents in brain, retina, heart and liver but not in RBC (Figure 2A). The direct supplementation with DHA increased total n-3 PUFA content in all studied tissues. More precisely (Figure 2B), total n-3 PUFA content was higher in brain and retina in the DL group, in comparison to the VO group and this increase reached the same level than in both groups directly supplemented with DHA. In the heart, the total n-3 PUFA content was higher for the DL+DHA group than for the VO+DHA group.

**Figure 3:**
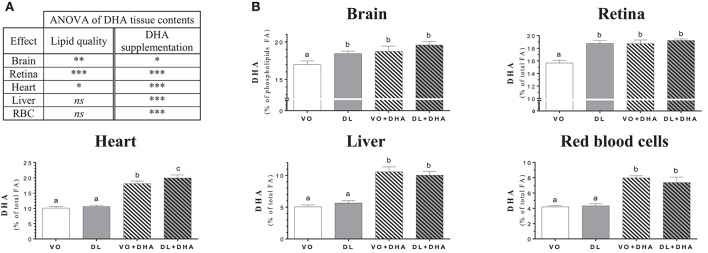
**(A)** Overall lipid quality (LQ) effect and DHA supplementation (S) effect of the docosahexaenoic acid (DHA) proportions in brain, retina, heart, liver and red blood cells (RBC) of rats fed the vegetable oil blend diet (VO) or the vegetable oil and dairy lipid blend diet (DL); not supplemented or supplemented with 0.5% DHA (VO+DHA and DL+DHA). The significance of the two effects was assessed by two-ways ANOVA (^*^p < 0.05, ^**^p < 0.01, ^***^p < 0.001). ^*^Represents globally an increase in favor of the DL groups for the LQ effect, and in favor of the lots supplemented with DHA for the S effect. *ns*, not significant. **(B)** Proportions of DHA in tissues. Values are mean ± SEM. Different superscript letters indicate significant differences for multiple comparisons (*p* < 0.05).

Concerning DHA tissue contents (Figure 3), if we first consider the overall effects (Figure 3A), rats fed DL diets had higher DHA contents in brain, retina and heart but not in liver and RBC. The direct supplementation with DHA, increased DHA content in all studied tissues. More precisely (Figure 3B), the DHA content was similarly affected by the diets than the total n-3 LCPUFA status, as DHA was the major n-3 LCPUFA found in tissues. The DHA content was higher in brain and retina in the DL group, in comparison to the VO group and this increase reached the same DHA level than in both groups directly supplemented with DHA. In the heart, the total n-3 LCPUFA content was higher for the DL+DHA group than for the VO+DHA group.

**Figure 4:**
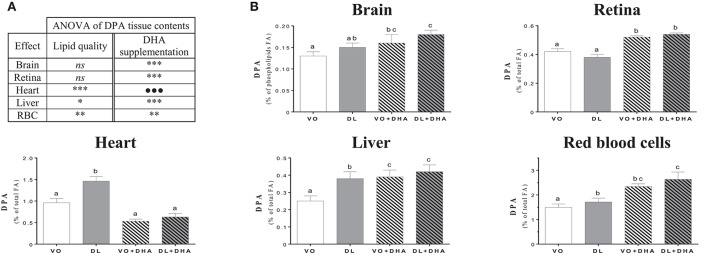
**(A)** Overall lipid quality (LQ) effect and DHA supplementation (S) effect of the n-3 docosapentaenoic acid (n-3 DPA) proportions in brain, retina, heart, liver and red blood cells (RBC) of rats fed the vegetable oil blend diet (VO) or the vegetable oil and dairy lipid blend diet (DL), not supplemented or supplemented with 0.5% DHA (VO+DHA and DL+DHA). The significance of the two effects was assessed by two-ways ANOVA (^*^p < 0.05, ^**^p < 0.01, ^***^p < 0.001). ^*^Represents globally an increase in favor of the DL groups for the LQ effect, and in favor of the lots supplemented with DHA for the S effect. *ns*, not significant. **(B)** Proportions of DHA in tissues. Values are mean ± SEM. Different superscript letters indicate significant differences for multiple comparisons (*p* < 0.05).

Concerning n-3 DPA contents (Figure 4), if we first consider the overall effects (Figure 4A), rats fed DL diets had higher n-3 docosapentaenoic acid (n-3 DPA) contents in heart, liver and RBC but not in brain and retina conversely than found for DHA. Moreover, n-3 DPA content increased with DHA supplementation in all tissues except in the heart. More precisely (Figure 4B), the n-3 DPA content was higher in the heart, liver and RBC of DL-fed rats compared to the VO group but remained lower than in rats supplemented with DHA in liver and RBC. We underline a point of interest demonstrated by redundancy analysis approach: the n-3 DPA was the FA, which positively discriminated the most DL groups compared to VO groups (see Supplementary Material 3).

Concerning eicosapentaenoic acid (EPA), its content was also higher with DL diets in retina and liver compared to VO group but not in other tissues (see Supplementary Material 2, Tables 1–5). In the liver, the retroconversion of DHA toward EPA was estimated at 72.4% with no difference between DHA-supplemented groups.

By contrast with the interesting effect of DL diets on n-3 LCPUFA, no clear effect was observed on n-6 LCPUFA, except in retina where DL diets increased all n-6 polyunsaturated FA, except for the n-6 DPA (Table 2). The DHA supplementation decreased all the n-6 LCPUFA contents in all studied tissues.

**Table 2:** The n-6 polyunsaturated fatty acids composition in tissues of rats fed the four diets.

						**ANOVA**	
	**Fatty acids (%)**	**VO**	**DL**	**VO+DHA**	**DL+DHA**	**LQ**	**S**
Brain	ARA	11.04^ab^ ± 0.31	11.87^b^ ± 0.30	9.84^a^ ± 0.40	10.38^ab^ ± 0.39	.	∙
	AdA	3.04^a^ ± 0.17	3.04^a^ ± 0.16	1.76^b^ ± 0.09	1.85^b^ ± 0.10		∙∙∙
	n-6 DPA	0.54^a^ ± 0.06	0.50^a^ ± 0.05	0.14^b^ ± 0.02	0.14^b^ ± 0.02		∙∙∙
	∑ n-6	15.73^a^ ± 0.44	16.57^a^ ± 0.42	12.92^b^ ± 0.38	13.76^ab^ ± 0.40	.	∙∙
Retina	ARA	7.72^a^ ± 0.27	9.09^b^ ± 0.13	7.58^a^ ± 0.14	7.71^a^ ± 0.11	^***^	∙∙∙
	AdA	1.31^a^ ± 0.04	1.61^b^ ± 0.10	0.97^c^ ± 0.04	1.04^c^ ± 0.05	^**^	∙∙∙
	n-6 DPA	0.28^a^ ± 0.02	0.30^a^ ± 0.01	tr	0.10^b^ ± 0.01	^***^	∙∙∙
	∑ n-6	12.36^ab^ ± 0.35	13.07^a^ ± 0.09	11.25^c^ ± 0.13	11.68^bc^ ± 0.20	^*^	∙∙∙
Heart	ARA	22.62^a^ ± 0.26	21.32^a^ ± 0.47	13.83^b^ ± 0.35	13.71^b^ ± 0.24		∙∙∙
	AdA	0.65^a^ ± 0.06	0.80^a^ ± 0.06	0.08^b^ ± 0.02	0.09^b^ ± 0.02		∙∙∙
	n-6 DPA	0.78^a^ ± 0.08	0.73^a^ ± 0.05	tr	tr		∙∙∙
	∑ n-6	37.18^a^ ± 0.29	37.58^b^ ± 0.51	27.91^c^ ± 0.53	28.40^c^ ± 0.45	^*^	∙∙∙
Liver	ARA	13.43^a^ ± 0.82	13.48^a^ ± 0.56	9.07^b^ ± 0.67	8.01^b^ ± 0.86		∙∙∙
	AdA	0.15^a^ ± 0.02	0.17^a^ ± 0.02	0.06^b^ ± 0.01	0.06^b^ ± 0.01	.	∙∙
	n-6 DPA	0.15^a^ ± 0.02	0.16^a^ ± 0.02	0.02^b^ ± 0.01	0.01^b^ ± 0.01		∙∙∙
	∑ n-6	21.5 ± 1.05	23.23 ± 0.81	18.93 ± 0.92	19.14 ± 1.18		∙
RBC	ARA	25.57^a^ ± 0.70	24.38^b^ ± 0.88	17.69^c^ ± 0.40	17.82^c^ ± 1.07	.	∙∙∙
	AdA	1.24^a^ ± 0.08	1.24^a^ ± 0.09	0.42^b^ ± 0.05	0.60^b^ ± 0.11		∙∙∙
	n-6 DPA	0.59^a^ ± 0.05	0.52^a^ ± 0.04	0.10^b^ ± 0.04	0.23^b^ ± 0.06		∙∙∙
	∑ n-6	34.39^a^ ± 0.71	34.14^a^ ± 0.71	26.63^b^ ± 0.39	27.73^b^ ± 0.86		∙∙∙

*Total fatty acid proportions of linoleic acid (LA, C18:2 n-6), arachidonic acid (ARA, C20:4 n-6), adrenic acid (AdA, C22:4 n-6), n-6 docosapentaenoic acid (n-6 DPA, C22:5 n-6) and total n-6 long-chain polyunsaturated fatty acids (∑ n-6), in tissues of rats fed the vegetable oil blend diet (VO) or the vegetable oil and dairy lipid blend diet (DL), not supplemented or supplemented with 0.5% DHA (VO+DHA and DL+DHA). For the brain, the fatty acid composition from phospholipids is presented. Values are mean ± SEM*.

^*^Indicates an increase and • a decrease in favor of the DL groups for the lipid quality effect (LQ) and in favor of the groups supplemented with DHA for the DHA supplementation effect (S) (^*^p < 0.05, ^**^p < 0.01, ^***^p < 0.001 and ^·^0.1 < p < 0.05).

### Dairy lipids saved LCPUFA precursors α-linolenic acid (ALA) and linoleic acid (LA)

**Figure 5:**
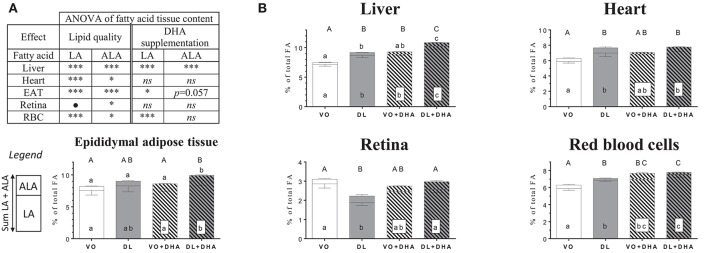
**(A)** Overall lipid quality (LQ) effect and DHA supplementation (S) effect of linoleic acid (LA) and α-linolenic acid (ALA) proportions in brain, retina, heart, liver and red blood cells (RBC) of rats fed the vegetable oil blend diet (VO) or the vegetable oil and dairy lipid blend diet (DL), not supplemented or supplemented with 0.5% DHA (VO+DHA and DL+DHA) (^*^p < 0.05, ^**^p < 0.01, ^***^p < 0.001). ^*^Represents globally an increase and • a decrease in favor of the DL groups for the LQ effect, and in favor of the lots supplemented with DHA for the S effect. *ns*, not significant. **(B)** Proportions of LA (bottom), ALA (top) and sum ALA+LA (whole histogram, uppercase letters) in tissues. Values are mean ± SEM. Different superscript letters indicate significant differences for multiple comparisons (*p* < 0.05).

We have also considered the effects of the different diets on the n-3 LCPUFA precursor ALA and n-6 LCPUFA precursor LA (Figure 5). First, if we consider the overall effects (Figure 5A), we showed that DL diets increased both ALA and LA amounts in all studied tissues, except in brain and retina. Specifically, in the retina, DL diets decreased the LA proportion while increasing the ALA proportion. In the brain, only the proportion of LA was increased with DL diets, and ALA was not detected (Supplementary Material 2, Table 1). The DHA supplemented diets increased LA in the liver, RBC and EAT, and ALA in liver only.

If we compare now each group (Figure 5B), the ALA content was higher in the liver in rats fed DL compared to the VO group, reaching the same level than the VO+DHA group. Moreover, ALA levels were higher in the DL+DHA group than in other groups. We showed that the sum of ALA+LA was higher in DL group compared to the VO group in liver, heart, and RBC, reaching the same level than VO+DHA group. In contrast, the retina was the only tissue where the sum ALA+LA decreased in DL group. DL+DHA group exhibited a higher sum of ALA+LA than other groups in the liver, RBC and EAT. For all groups in all tissues, LA contents and the sum ALA+LA showed the same effects.

Finally, in the liver, the Δ-6 desaturase specific activity as well as the FA desaturase 2 mRNA relative expression (*Fads2*) did not change whatever the groups (Supplementary Material 4, Figure 1).

### Dairy lipids enriched fatty acid composition of tissues

DL diets contained 50% (*w/w*) of dairy lipids from butterfat, which provides specific FA absent in vegetable oils: 4.8% short- and medium-chain fatty acids (SMCFA) (C4:0 to C12:0), 5.5% of myristic acid (C14:0) and 2.1% of branched-chain FA (BFA), odd FA (C15:0) (Table 1).

The FA composition of the epididymal adipose tissue (EAT) was used as a marker of the long-term storage of FA provided by the diets (Table 3). As expected, the specific FA present in dairy lipids increased in DL and DL+DHA groups compared to VO and VO+DHA groups. More precisely, the content in C12:0 and C15:0 increased in EAT with DL diets. The content in C10:0, C14:1 n-5, C16:0 iso, C17:0 iso, and C17:0 ante-iso were quantifiable in both DL groups and only detected as trace in VO and VO+DHA groups.

**Table 3:** Total fatty acids composition of epididymal adipose tissue of rats fed the four diets.

**Total fatty acids (%)**	**VO**	**DL**	**VO+DHA**	**DL+DHA**	**ANOVA**	
					**LQ**	**S**
C10:0	tr	0.20^b^ ± 0.02	tr	0.22^b^ ± 0.03	^***^	
C12:0	0.18^a^ ± 0.06	0.61^b^ ± 0.06	0.15^a^ ± 0.04	0.68^b^ ± 0.08	^***^	
C14:0	1.21^a^ ± 0.13	3.53^b^ ± 0.17	1.19^a^ ± 0.13	3.70^b^ ± 0.17	^***^	
C15:0	0.12^a^ ± 0.02	0.39^b^ ± 0.05	0.12^a^ ± 0.01	0.41^b^ ± 0.03	^***^	
C16:0	22.57 ± 1.78	24.54 ± 1.70	23.71 ± 1.26	25.51 ± 1.30	^**^	.
C18:0	1.79^a^ ± 0.17	2.46^b^ ± 0.29	2.28^b^ ± 0.30	3.24^c^ ± 0.26	^***^	^***^
∑ SFA	25.81^a^ ± 2.00	31.35^b^ ± 1.57	27.39^a^ ± 1.58	33.35^b^ ± 1.31	^***^	^**^
C16:1 n-9	0.50^ab^ ± 0.06	0.51^b^ ± 0.04	0.44^a^ ± 0.04	0.44^a^ ± 0.04		∙∙∙
C18:1 n-9	56.74^a^ ± 2.38	46.93^b^ ± 1.81	55.98^a^ ± 1.66	46.47^b^ ± 1.33	∙∙∙	
C20:1 n-9	0.13 ± 0.02	0.12 ± 0.02	0.12 ± 0.01	0.11 ± 0.02	.	
∑ n-9	57.38^a^ ± 2.37	47.56^b^ ± 1.77	56.55^a^ ± 1.66	47.02^b^ ± 1.33	∙∙∙	
C16:1 n-7	4.17^a^ ± 0.76	5.92^b^ ± 1.10	3.78^a^ ± 0.68	4.60^a^ ± 0.64	^***^	∙∙
C18:1 n-7	4.21^a^ ± 0.57	4.98^b^ ± 0.42	3.26^ac^ ± 0.28	3.74^c^ ± 0.31	^***^	∙∙∙
∑ n-7	8.38^a^ ± 1.22	10.90 ± 1.48	7.03^ab^ ± 0.73	8.34^a^ ± 0.87	^***^	∙∙∙
C18:2 n-6 (LA)	7.55^a^ ± 0.71	8.29^ab^ ± 0.92	7.85^a^ ± 0.44	9.04^b^ ± 0.68	^***^	^*^
C20:4 n-6 (ARA)	tr	tr	tr	tr		
∑ n-6	7.61^a^ ± 0.74	8.33^ab^ ± 0.93	7.88^a^ ± 0.43	9.08^b^ ± 0.69	^***^	.
C18:3 n-3 (ALA)	0.64^a^ ± 0.07	0.74^a^ ± 0.09	0.75^a^ ± 0.07	0.88^b^ ± 0.10	^***^	^***^
C22:6 n-3 (DHA)	tr	tr	0.21^b^ ± 0.11	0.21^b^ ± 0.10		^***^
∑ n-3	0.64^a^ ± 0.07	0.75^a^ ± 0.10	0.96^b^ ± 0.14	1.09^b^ ± 0.18	^*^	^***^
C14:1 n-5	tr	0.29^c^ ± 0.03	tr	0.36^b^ ± 0.02	^***^	.
C16:0 iso	tr	0.11^b^ ± 0.01	tr	0.10^b^ ± 0.02	^***^	
C17:0 iso	tr	0.14^b^ ± 0.03	tr	0.13^b^ ± 0.04	^***^	
C17:0 ante	tr	0.13^b^ ± 0.02	tr	0.14^b^ ± 0.02	^***^	
∑ others	tr	1.11^b^ ± 0.07	tr	1.12^b^ ± 0.08	^***^	

*Rats fed the vegetable oil blend diet (VO) or the vegetable oil and dairy lipid blend diet (DL), not supplemented or supplemented with 0.5% DHA (VO+DHA and DL+DHA). Values are mean ± SEM*.

^*^Indicates an increase and • a decrease in favor of the DL groups for the lipid quality effect (LQ) and in favor of the groups supplemented with DHA for the DHA supplementation effect (S) (^*^p < 0.05, ^**^p < 0.01, ^***^p < 0.001, ^·^0.1 < p < 0.05).

In addition, myristic acid was present in all diets but mainly in DL diets, so myristic acid was higher in EAT of DL groups compared to VO groups. A similar observation was made in the liver, as in EAT for myristic acid and C15:0 (see Supplementary Material 2, Table 3).

### Dairy lipids increased plasma cholesterol

DL diets contained 6.6 mg of cholesterol/100 g of diet, whereas cholesterol was absent in VO diets. The DHA supplementation let to an overall decrease in total cholesterol, HDL cholesterol and non-HDL cholesterol. However, no significant effect was found in total cholesterol/HDL cholesterol ratio (Table 4). Plasmatic total cholesterol and non-HDL cholesterol were higher only in DL group compared to the three other groups. The supplementation of DL diet with DHA maintained the total cholesterol amount at the same level than both VO and VO+DHA groups.

**Table 4:** Plasma concentration of total cholesterol, HDL cholesterol and non-HDL cholesterol.

**mg cholesterol/dL of plasma**	**VO**	**DL**	**VO+DHA**	**DL+DHA**	**ANOVA**		
					**LQ**	**S**	LQ^*^S
Total	79.85 ± 4.1^a^	94.56 ± 4.13^b^	70.94 ± 2.20^ac^	65.60 ± 3.43^c^	^*^	^***^	^*^
HDL	52.80 ± 3.10^a^	52.76 ± 3.00^a^	44.87 ± 1.70^ab^	37.90 ± 3.02^b^	ns	^***^	ns
Non-HDL	27.06 ± 2.29^a^	41.80 ± 2.81^b^	26.07 ± 1.51^a^	27.73 ± 1.59^a^	^***^	∙∙	^*^
Total/HDL	1.54 ± 0.06^a^	1.83 ± 0.07^c^	1.59 ± 0.04^ab^	1.77 ± 0.08^bc^	^***^	ns	ns

*Rats fed the vegetable oil blend diet (VO) or the vegetable oil and dairy lipid blend diet (DL), not supplemented or supplemented with 0.5% DHA (VO+DHA and DL+DHA). Values are mean ± SEM*.

^*^Indicates an increase and ∙ a decrease in favor of the DL groups for the lipid quality effect (LQ) and in favor of the groups supplemented with DHA for the DHA supplementation effect (S) (^*^p < 0.05, ^***^p < 0.001). ns: not significant.

## Discussion

This study addressed the capacity of a partial incorporation of dairy lipids (DL) in diets to modulate the n-3 long-chain polyunsaturated fatty acids (LCPUFA) status in tissues, comparatively to a vegetable oils blend based diet (VO). Both diets were not supplemented or supplemented with 0.5% DHA (DL+DHA and VO+DHA).

The main finding of our study is that DL diet increased DHA levels in brain and retina at the same levels than DHA-supplemented diets. Moreover, both DL and DL+DHA diets increased also α-linolenic acid (ALA) amount in the liver and increased linoleic acid (LA) amount in all studied tissues. The effect observed here on DHA status is consistent with several studies, obtained in different specific conditions. Precisely, dairy lipids increased the brain DHA status compared to palm oil (27) in a model of ALA deficiency fed diets containing 1.5 or 2.3% ALA of total FA (*w/w*). Under equivalent conditions of ALA deficiency (28), the same authors confirmed that the amount of DHA in the brain increased in rats fed butter blend diet containing 2.3% ALA compared to 1.5%, but showed no additional increase of DHA with rats fed pure rapeseed diet containing 8.3% ALA. This latter consideration led us to choose the 2.3% ALA value for our present experimental diets. Another study recently showed that dairy lipid-enriched diet also increased DHA status in the brain of young and adult mice, beginning the diets on the first day of gestation until offspring's adulthood and with a different LA/ALA ratio than in our study (7). Finally, the increase of brain DHA status presented here is significant but smaller than in other studies, and was observed in brain phospholipids (not in brain total FA, data not shown). There was no deficiency before the experiment under our conditions, all rats were fed experimental diets from weaning with the same proportion of 2.3% ALA in diets. Then, both lipid bases of the diets had the same LA/ALA ratio of 5, closed to recommendations in humans (32), to overcome the known effects of the LA/ALA ratio on the LCPUFA metabolism (28, 42, 51, 52).

A first mechanistic hypothesis to explain the increase of DHA in brain and retina is that the DL diet could act on the n-3 LCPUFA conversion pathway. Indeed, it has been reported that the expression of *Elovl2* and *Fads2* genes, coding, respectively, for the elongase-2 and the Δ-6 desaturase enzymes, were increased with high butter blend diets in ALA (2.3%) compared to vegetable oils blend diets, lower in ALA (1.3%) (27). In our study, no effect of the diet was found on the Δ-6 desaturase activity and expression, with the limitation of measurements performed at the fasting state. This result is not surprising as we used conditions where the conversion pathway was highly activated. Moreover, another possibility to explain the increase of n-3 LCPUFA with DL diets is the specific presence of myristic acid (C14:0) in DL diet. It has been reported that this FA increased dose-dependently and specifically the activity of Δ-6 and Δ-5 desaturases in primary rat hepatocytes (53). And we previously demonstrated in our laboratory that C14:0 can improve the n-3 LCPUFA content (36, 54, 55). In another way, the Δ-6 desaturase has a better affinity for ALA than for LA (56), and could also explain that only n-3 LCPUFA and not n-6 LCPUFA increased in our study. However, it has been shown that the content of LCPUFA in tissue is less influenced by the regulation of the desaturase and elongase genes, than by the substrate level (51).

A second possibility to explain the increase in the n-3 LCPUFA content is the presence of saturated FA in DL diets, and especially short- and medium-chain FA (SMCFA, ≤ 12 carbons). The high mitochondrial β-oxidation of SMCFA could partially spare ALA and LA from β-oxidation and could orient ALA toward the n-3 LCPUFA conversion pathway instead of the β-oxidation pathway (2, 57). Indeed, as the oxidation of FA is well correlated with the number of carbon and the number of double bonds (58), ALA and LA are also good substrates for the β-oxidation, as well as oleic acid (59), and the oxidation rate of FA is reported as follows: SMCFA >> ALA > oleic acid > LA (60). Consequently, in our study, the consumption of SMCFA with DL diets could prevent part of the β-oxidation of LCPUFA precursors ALA and LA (61).

A third hypothesis to partly explain the increase in n-3 LCPUFA in tissues with DL diets is the lower level of oleic acid in DL diets than in VO diets. Indeed, oleic acid is also a substrate of Δ-6 desaturase and could compete with ALA and LA. In our study, oleic acid was higher in VO diets. However, the possible competition of oleic acid for the Δ-6 desaturase appears unlikely, since no lower content of n-6 LCPUFA was observed with VO diets (62).

The second major finding of our study is that n-3 docosapentaenoic acid (n-3 DPA) levels increased in heart, liver, and RBC with DL diet, by contrast with brain and retina where DHA status increased. The n-3 DPA is one intermediate compound in the n-3 LCPUFA conversion pathway. Compared to EPA, n-3 DPA is downstream in the n-3 LCPUFA conversion pathway, so it could be a better precursor of DHA in tissues. In the present study, brain n-3 DPA content appears to remain relatively stable within the different diets. Moreover, only direct DHA supplementation resulted in a significant slight increase in n-3 DPA in the brain, possibly originating from the retroconversion of DHA to EPA, which is quickly converted into n-3 DPA or released from the brain into the bloodstream. Some authors hypothesized that n-3 DPA present in RBC could be a good predictor for brain DHA, showing a correlation (*R*^2^ = 0.478) between the n-3 DPA present in the RBC and the brain DHA (27). We found a similar correlation in the present study (*R*^2^ = 0.464) by using data from VO and DL groups in the model (data not shown), but this predictor hypothesis remains unclear in the absence of metabolic plausibility. Finally, the mechanisms of transport, use and conversion, specific to n-3 DPA are still poorly known and require further research, even if some studies report that n-3 DPA may react more like DHA than EPA for these aspects (2, 63).

The metabolism of n-3 DPA is poorly studied yet, due to its lack of commercial availability at high level of purity to realize *in vivo* studies. However, recent *in vitro* and *in vivo* studies showed that n-3 DPA could have some specific physiological effect, and particularly to prevent cardiovascular diseases by decreasing cholesterol, triglycerides synthesis and inflammation (2, 11, 63–65). Interestingly, our results showed that for both DL diets, n-3 DPA is the most discriminated FA in the heart. The potential synergic effect of n-3 DPA combined with DL on cardiovascular prevention may be investigated in future studies. Moreover, some recent cohort studies suggest that n-3 DPA could be the more potent n-3 LCPUFA to prevent some pathologies like metabolic syndrome, depression or lung dysfunction (4, 66, 67). For further *in vivo* studies, we need to obtain highly purified n-3 DPA, as already proposed by some laboratories (68).

Concerning cholesterol, some studies showed that dairy lipid-enriched diets could affect the amount of cholesterol in some tissues (69, 70). As a result, we showed in this study that plasma cholesterol increased only with DL group, and this is interesting as cholesterol is important during growth and was not provided by VO diets. Moreover, our results showed interestingly that plasma cholesterol was maintained at similar levels as the VO non-supplemented diet when DL diet was supplemented with DHA, while it was not significantly decreased when the VO diet was supplemented with DHA. Moreover, plasma cholesterol decreased with DL+DHA group compared to DL group, and at the same level than VO+DHA group. This observation is important because infant formula including DHA will be mandatory in a few years in Europe (23).

Obviously, the comparison between rats and humans remains a question and a limit of the putative extrapolation of the results of the present study (71). However, in the present work, we could compare the age of our male rats fed from weaning to 9 weeks of age with an interval of 6 months to 14 years old in humans (72), a period where the consumption of dairy products occurs.

In conclusion, our results showed that n-3 LCPUFA content was positively influenced by dairy lipids and differently according to the tissues. DL diet increased DHA in brain and retina at the same levels than groups supplemented with DHA; and increased n-3 DPA in heart, RBC and liver. This accretion of n-3 LCPUFA suggests interesting roles of specific FA or other components of dairy lipids.

## Author contributions

Under the supervision of PL and DC, GD completed these studies as part of his Ph.D. thesis. GD, PL, CB, PLR, DC, and VR contributed to the conception and design of the project. GD, AS, and DC performed experimentation. GD conducted the literature search, the acquisition and analysis of data, and wrote the first version of the manuscript. PL, VR, DC, CB, and PLR contributed to the revision of the manuscript. All authors read and approved the final work.

### Conflict of interest statement

CB and PLR are employed by Lactalis group. GD, DC, AS, VR and PL received financial support from Lactalis group.

## Abbreviations

ALA: n-3 α-linolenic acid
DHA: n-3 docosahexaenoic acid
DL: blend of vegetable oils and dairy lipids
n-3 DPA: n-3 docosapentaenoic acid
EAT: epididymal adipose tissue
EPA: n-3 eicosapentaenoic acid
FA: fatty acid(s)
Fads2: fatty acids desaturase 2 gene
HDL: high-density lipoprotein(s)
LA: n-6 linoleic acid
LCPUFA: long-chain polyunsaturated fatty acid(s)
PCA: principal component analysis
RBC: red blood cells
RDA: redundancy analysis
SMCFA: short- and medium-chain fatty acids
VO: blend of vegetable oils.
