# Supplementary material for: Incorporation of Dairy Lipids in the Diet Increased Long-Chain Omega-3 Fatty Acids Status in Post-weaning Rats
Source: Front Nutr. 2018 May 23;5:42. doi: 10.3389/fnut.2018.00042 (PMC5974923; doi:10.3389/fnut.2018.00042)
Supplement: Supplementary file 1 [file Table_1.PDF]

Supplementary Material 1

Dairy lipids enriched diet increased Omega-3 status in post-weaning rats.

Gaetan Drouin[1], Daniel Catheline[1], Anaëlle Sinquin[1], Charlotte Baudry[2], Pascale Le Ruyet[2], Vincent Rioux[1], Philippe Legrand[1*]

*** Correspondence:** Corresponding Author: philippe.legrand@agrocampus-ouest.fr

1 Supplementary material 1 - Table 1: Organs weight at the end of the experimentation

Organs weight after 6 weeks of diets

	VO	DL	VO+DHA	DL+DHA
**Liver**	3.06 *±0.06*	3.07 *±0.06*	3.12 *±0.07*	3.12 *±0.11*
**Brain**	0.41[a] *±0.01*	0.47[b] *±0.01*	0.43[ab] *±0.01*	0.42[ab] *±0.01*
**Heart**	0.33 *±0.01*	0.35 *±0.01*	0.35 *±0.01*	0.34 *±0.01*

Organs weight of rats after 6 weeks of diets are presented (n = 8 / group for rats supplemented with DHA, n = 16 / group for rats not supplemented for 6 weeks). Results are mean ± SEM. A linear mixed model with two fixed factors (LQ: Lipid Quality, S: DHA supplementation) adjusted to the date of study is realized. The linear model is followed by a post-hoc test of Tukey-Kramer adjusted by false discovery rate method. Two different letters indicate significantly different values.

2 Supplementary material 1 - Figure 1: Rats body weight gain (A), food (B) and water consumption (C).

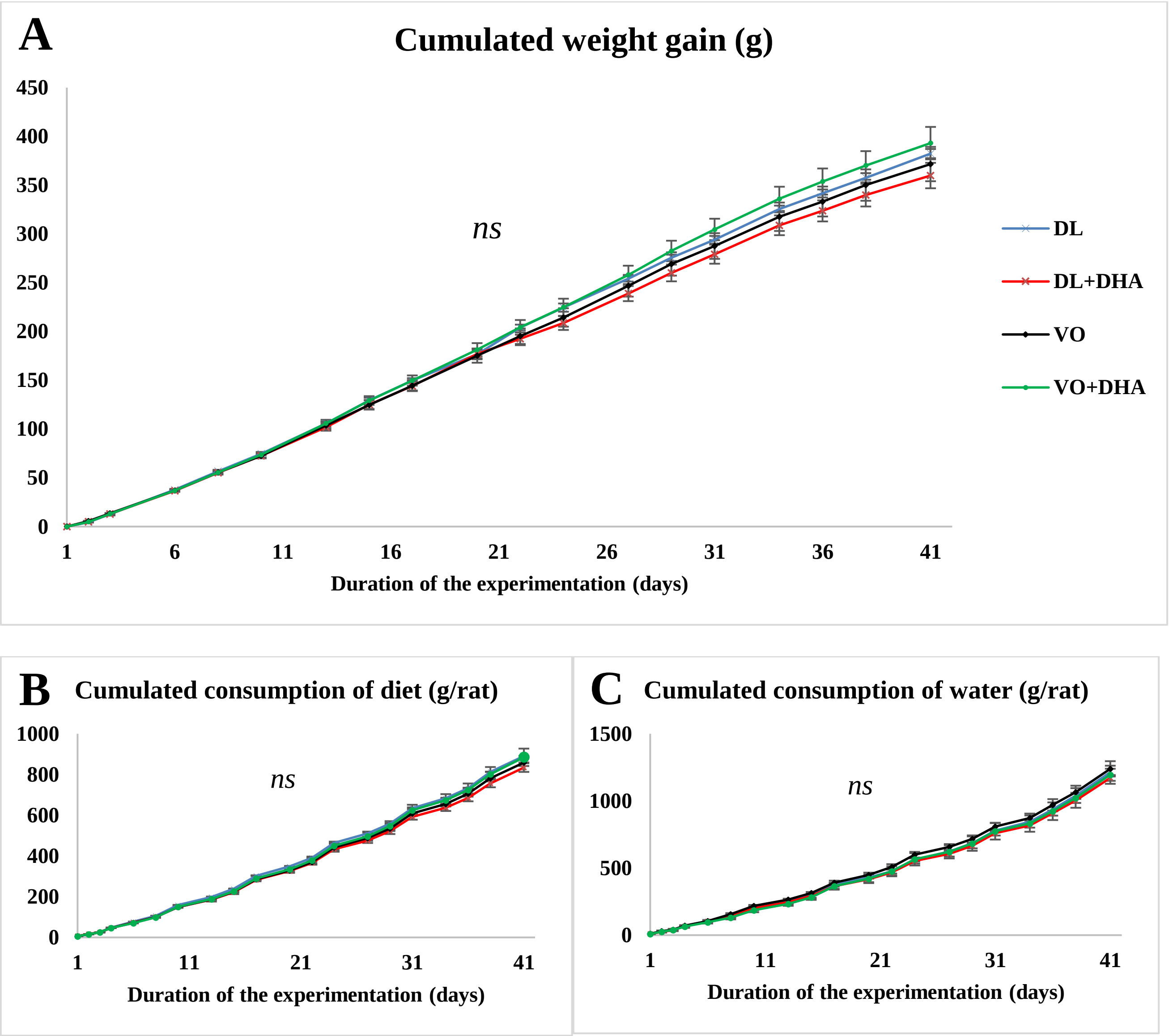

Cumulated weigh gain, diet consumption and water consumption of rats after 6 weeks of diets are presented as mean ± SEM (n = 8 / group for rats supplemented with DHA, n = 16 / group for rats not supplemented). A linear mixed model for repeated measures with two fixed adjusted to the date of study is realized. The linear model is followed by a post-hoc test of Tukey-Kramer adjusted by false discovery rate method. *ns*: non significant.
