# Supplementary material for: Incorporation of Dairy Lipids in the Diet Increased Long-Chain Omega-3 Fatty Acids Status in Post-weaning Rats
Source: Front Nutr. 2018 May 23;5:42. doi: 10.3389/fnut.2018.00042 (PMC5974923; doi:10.3389/fnut.2018.00042)
Supplement: Supplementary file 2 [file Table_2.PDF]

Supplementary Material 2

Dairy lipids enriched diet increased Omega-3 status in post-weaning rats.

Gaetan Drouin[1], Daniel Catheline[1], Anaëlle Sinquin[1], Charlotte Baudry[2], Pascale Le Ruyet[2], Vincent Rioux[1], Philippe Legrand[1*]

*** Correspondence:** Corresponding Author: philippe.legrand@agrocampus-ouest.fr

1 Supplementary material 2 - Table 1: Brain fatty acids composition of phospholipids

2 Supplementary material 2 - Table 2: Retina fatty acids composition of total lipids

3 Supplementary material 2 - Table 3: Liver fatty acids composition of total lipids

4 Supplementary material 2 - Table 4: Heart fatty acids composition of total lipids

5 Supplementary material 2 - Table 5: Red blood cells fatty acids composition of total lipids

<u>Legend for all Tables:</u>

The mass percentages of the identified fatty acids obtained by gas chromatography mass spectrophotometry are presented as mean ± SEM of rats fed the vegetable oil blend diet (VO) or the vegetable oil and dairy lipid blend diet (DL); not supplemented or supplemented with 0.5% DHA (VO+DHA and DL+DHA). A linear mixed model with two fixed factors (LQ: Lipid Quality, S: DHA supplementation) adjusted to the duration of the diets and the date of study was realized. A * represents globally an increase and ● a decrease in favor of the DL groups for the LQ effect, in favor of the lots supplemented with DHA for the S effect (* p <0.05, ** p <0.01; *** p <0.001; . 0.1 <p <0.05). The linear model was followed by a post-hoc test of Tukey-Kramer adjusted by false discovery rate method. Two different letters indicate significantly different values. Fatty acids with a proportion <0.1% are noted as trace (tr).

Supplementary material 2 - Table 1: Brain fatty acids composition of phospholipids

	VO	DL	VO+DHA	DL+DHA	ANOVA		
					LQ	S	LQ*S
C14:0	0.17 *±0.04*	0.15 *±0.02*	0.13 *±0.01*	0.16 *±0.02*			
C16:0	20.53^{ab} *±0.54*	19.54^{b} *±0.51*	22.25^{a} *±0.66*	19.74^{b} *±0.68*	••		
C18:0	21.08 *±0.48*	20.87 *±0.65*	23.11 *±0.57*	23.11 *±0.35*			
C20:0	0.27 *±0.02*	0.25 *±0.02*	0.22 *±0.03*	0.22 *±0.05*			
**Σ SFA**	**42.05^{ab}** ***±0.59***	**40.81^{b}** ***±0.78***	**45.71^{a}** ***±0.74***	**43.23^{ab}** ***±0.60***	••		
C16:1 n-9	0.15 *±0.02*	0.26 *±0.13*	0.10 *±0.01*	0.10 *±0.00*			
C18:1 n-9	20.04 *±0.80*	18.47 *±0.51*	20.21 *±0.74*	21.08 *±0.52*			
C20:1 n-9	1.29 *±0.11*	1.16 *±0.08*	1.14 *±0.19*	1.05 *±0.15*			
**Σ n-9**	**21.48** ***±0.82***	**19.89** ***±0.47***	**21.45** ***±0.89***	**22.23** ***±0.58***			
C16:1 n-7	0.36 *±0.01*	0.44 *±0.06*	0.37 *±0.02*	0.35 *±0.01*			
C18:1 n-7	4.26 *±0.09*	4.10 *±0.13*	3.82 *±0.21*	4.09 *±0.11*		·	
C20:1 n-7	0.45 *±0.04*	0.45 *±0.03*	0.47 *±0.06*	0.41 *±0.05*			*
**Σ n-7**	**5.07** ***±0.11***	**4.99** ***±0.13***	**4.67** ***±0.24***	**4.85** ***±0.15***		·	
C18:2 n-6	0.73 *±0.06*	0.76 *±0.06*	0.78 *±0.04*	0.96 *±0.10*	*		
C20:3 n-6	0.33^{a} *±0.02*	0.40^{b} *±0.02*	0.42^{c} *±0.02*	0.42^{c} *±0.03*	**	***	
C20:4 n-6	11.04^{ab} *±0.31*	11.87^{b} *±0.30*	9.84^{a} *±0.40*	10.38^{ab} *±0.39*	·	•	
C22:4 n-6	3.04^{a} *±0.17*	3.04^{a} *±0.16*	1.76^{b} *±0.09*	1.85^{b} *±0.10*		•••	
C22:5 n-6	0.54^{a} *±0.06*	0.50^{a} *±0.05*	0.14^{b} *±0.02*	0.14^{b} *±0.02*		•••	
**Σ n-6**	**15.73^{a}** ***±0.44***	**16.57^{a}** ***±0.42***	**12.92^{b}** ***±0.38***	**13.76^{ab}** ***±0.40***	·	••	
C22:5 n-3	0.13^{a} *±0.01*	0.15^{ab} *±0.01*	0.16^{bc} *±0.02*	0.18^{c} *±0.01*		***	
C22:6 n-3	16.98^{a} *±0.28*	18.43^{b} *±0.35*	18.73^{b} *±0.71*	19.58^{b} *±0.45*	**	*	
**Σ n-3**	**17.11^{a}** ***±0.49***	**18.58^{b}** ***±0.61***	**18.89^{b}** ***±0.58***	**19.76^{b}** ***±0.37***	**	*	

For legend, see page 1.

Supplementary material 2 - Table 2: Retina fatty acids composition of total lipids

	VO		DL		VO+DHA		DL+DHA		ANOVA		
									LQ	S	LQ*S
C12:0	0.17	±0.04	tr		0.14	±0.03	0.10	±0.01	•		
C14:0	0.60	±0.04	0.59	±0.06	0.54	±0.03	0.60	±0.02			
C15:0	0.15^{a}	±0.01	0.21^{b}	±0.02	0.16^{ab}	±0.01	0.19^{ab}	±0.01	***		
C16:0	25.15	±0.47	25.12	±0.19	25.93	±0.29	25.51	±0.23		·	
C18:0	21.67^{a}	±0.31	23.01^{b}	±0.20	22.64^{ab}	±0.41	22.86^{b}	±0.24	*		·
**Σ SFA**	**47.74**	**±0.65**	**49.02**	**±0.24**	**49.41**	**±0.55**	**49.25**	**±0.34**		·	
C16:1 n-9	0.37	±0.01	0.44	±0.07	0.37	±0.02	0.33	±0.02			
C18:1 n-9	18.64^{a}	±0.61	13.65^{b}	±0.28	15.25^{b}	±0.5	14.33^{b}	±0.14	•••	••	***
C20:1 n-9	0.36	±0.04	0.42	±0.05	0.44	±0.05	0.37	±0.05			
**Σ n-9**	**19.36^{a}**	**±0.60**	**14.51^{b}**	**±0.30**	**16.05^{b}**	**±0.50**	**15.03^{b}**	**±0.15**	•••	••	***
C16:1 n-7	0.85	±0.09	0.80	±0.05	0.78	±0.06	0.82	±0.06			
C18:1 n-7	3.12^{a}	±0.18	2.74^{ab}	±0.09	2.45^{b}	±0.06	2.48^{b}	±0.04		•••	·
C20:1 n-7	0.13	±0.03	0.23	±0.05	0.26	±0.05	0.18	±0.04			
**Σ n-7**	**4.10^{a}**	**±0.22**	**3.77^{ab}**	**±0.09**	**3.49^{b}**	**±0.09**	**3.49^{b}**	**±0.07**		••	
C18:2 n-6	2.86^{a}	±0.22	1.87^{b}	±0.14	2.47^{ab}	±0.12	2.57^{a}	±0.17	*		**
C20:3 n-6	0.19^{a}	±0.01	0.20^{ab}	±0.01	0.23^{bc}	±0.01	0.26^{c}	±0.01	·	***	
C20:4 n-6	7.72^{a}	±0.27	9.09^{b}	±0.13	7.58^{a}	±0.14	7.71^{a}	±0.11	***	•••	**
C22:4 n-6	1.31^{a}	±0.04	1.61^{b}	±0.10	0.97^{c}	±0.04	1.04^{c}	±0.05	**	•••	·
C22:5 n-6	0.28^{a}	±0.02	0.30^{a}	±0.01	tr		0.10^{b}	±0.01	***	•••	*
**Σ n-6**	**12.36^{ab}**	**±0.35**	**13.07^{a}**	**±0.09**	**11.25^{c}**	**±0.13**	**11.68^{bc}**	**±0.20**	*	•••	
C18:3 n-3	0.24	±0.03	0.35	±0.08	0.27	±0.02	0.40	±0.07	*		
C20:5 n-3	0.12^{a}	±0.01	0.16^{a}	±0.01	0.28^{b}	±0.02	0.40^{c}	±0.05	*	***	
C22:5 n-3	0.42^{a}	±0.02	0.38^{a}	±0.02	0.52^{b}	±0.01	0.54^{b}	±0.01		***	·
C22:6 n-3	15.66^{a}	±0.43	18.75^{b}	±0.47	18.73^{b}	±0.58	19.23^{b}	±0.25	***	***	**
**Σ n-3**	**16.44^{a}**	**±0.43**	**19.64^{b}**	**±0.49**	**19.81^{b}**	**±0.59**	**20.56^{b}**	**±0.24**	***	***	*

For legend, see page 1.

Supplementary material 2 - Table 3: Liver fatty acids composition of total lipids

	VO	DL	VO+DHA	DL+DHA	ANOVA LQ	ANOVA S	ANOVA LQ*S
C14:0	0.57^{a} *±0.05*	0.95^{b} *±0.05*	0.45^{a} *±0.05*	0.85^{b} *±0.10*	***		
C15:0	0.14^{a} *±0.04*	0.34^{b} *±0.03*	0.13^{a} *±0.01*	0.25^{b} *±0.01*	***		
C16:0	23.84 *±0.69*	23.18 *±0.56*	24.53 *±0.21*	24.25 *±0.97*			
C18:0	12.61 *±0.73*	12.21 *±0.48*	13.71 *±1.05*	11.70 *±0.72*	•		
**Σ SFA**	**37.17^{ab}** ***±0.42***	**36.68^{b}** ***±0.47***	**38.83^{a}** ***±0.98***	**37.05^{ab}** ***±0.72***	·	·	
C16:1 n-9	0.50 *±0.04*	0.47 *±0.03*	0.63 *±0.06*	0.51 *±0.06*			
C18:1 n-9	29.78^{a} *±1.13*	26.24^{ab} *±0.99*	25.05^{b} *±2.37*	25.74^{ab} *±1.05*		••	
**Σ n-9**	**30.29** ***±1.17***	**26.71** ***±1.01***	**25.67** ***±2.41***	**26.25** ***±1.09***		•	
C16:1 n-7	2.07^{a} *±0.30*	2.77^{b} *±0.22*	1.51^{a} *±0.09*	2.17^{a} *±0.29*	***	•	
C18:1 n-7	3.22^{a} *±0.23*	3.78^{ab} *±0.13*	2.42^{a} *±0.06*	2.97^{b} *±0.16*	***	•••	
**Σ n-7**	**5.28^{a}** ***±0.37***	**6.56^{c}** ***±0.32***	**3.93^{b}** ***±0.11***	**5.14^{ab}** ***±0.44***	***	•••	
C18:2 n-6	7.15^{a} *±0.30*	8.67^{b} *±0.36*	8.92^{b} *±0.27*	10.25^{c} *±0.52*	***	***	
C18:3 n-6	0.18 *±0.02*	0.18 *±0.01*	0.15 *±0.02*	0.14 *±0.02*			
C20:2 n-6	0.16 *±0.01*	0.13 *±0.01*	0.16 *±0.03*	0.16 *±0.02*			
C20:3 n-6	0.29^{a} *±0.04*	0.44^{b} *±0.05*	0.54^{c} *±0.04*	0.51^{c} *±0.06*	·	***	·
C20:4 n-6	13.43^{a} *±0.82*	13.48^{a} *±0.56*	9.07^{b} *±0.67*	8.01^{b} *±0.86*		•••	
C22:4 n-6	0.15^{a} *±0.02*	0.17^{a} *±0.02*	0.06^{b} *±0.01*	0.06^{b} *±0.01*	·	••	
C22:5 n-6	0.15^{a} *±0.02*	0.16^{a} *±0.02*	0.02^{b} *±0.01*	0.01^{b} *±0.01*		•••	
**Σ n-6**	**21.5** ***±1.05***	**23.23** ***±0.81***	**18.93** ***±0.92***	**19.14** ***±1.18***		•	
C18:3 n-3	0.34^{a} *±0.04*	0.50^{b} *±0.05*	0.37^{ab} *±0.03*	0.60^{c} *±0.06*	***	***	
C20:5 n-3	0.12^{a} *±0.01*	0.28^{b} *±0.03*	1.32^{c} *±0.11*	1.38^{c} *±0.13*	**	***	
C22:5 n-3	0.25^{a} *±0.03*	0.38^{b} *±0.04*	0.39^{c} *±0.04*	0.42^{c} *±0.04*	*	***	
C22:6 n-3	5.05^{a} *±0.30*	5.66^{a} *±0.37*	10.57^{b} *±0.78*	10.02^{b} *±0.59*		***	
**Σ n-3**	**5.75^{a}** ***±0.34***	**6.82^{a}** ***±0.43***	**12.64^{b}** ***±0.84***	**12.42^{b}** ***±0.77***	***	***	

For legend, see page 1

Supplementary material 2 - Table 4: Heart fatty acids composition of total lipids

	VO		DL		VO+DHA		DL+DHA		ANOVA		
									LQ	S	LQ*S
C14:0	0.26[a]	*±0.03*	0.32[b]	*±0.03*	0.25[ab]	*±0.02*	0.33[b]	*±0.03*	**		
C15:0	0.02[a]	*±0.01*	0.15[b]	*±0.02*	0.10[ab]	*±0.01*	0.14[b]	*±0.01*	***	*	*
C16:0	12.84[a]	*±0.29*	12.39[b]	*±0.34*	14.19[c]	*±0.28*	13.74[c]	*±0.20*	•••	***	
C18:0	21.76	*±0.34*	20.92	*±0.25*	21.54	*±0.26*	21.65	*±0.31*	·		
**Σ SFA**	**34.88[a]**	***±0.58***	**33.78[b]**	***±0.51***	**36.09[a]**	***±0.38***	**35.86[a]**	***±0.43***	•	***	
C16:1 n-9	0.23	*±0.04*	0.15	*±0.01*	0.12	*±0.01*	0.13	*±0.02*			
C18:1 n-9	10.78	*±0.36*	10.07	*±0.47*	11.94	*±0.81*	9.66	*±0.76*	••		
C20:1 n-9	0.24	*±0.02*	0.22	*±0.02*	0.22	*±0.04*	0.20	*±0.03*			
C20:3 n-9	0.30[a]	*±0.03*	0.29[a]	*±0.03*	0.12[b]	*±0.02*	0.11[b]	*±0.02*		•••	
**Σ n-9**	**11.55**	***±0.36***	**10.74**	***±0.47***	**12.40**	***±0.83***	**10.10**	***±0.78***	••		
C16:1 n-7	0.55	*±0.07*	0.60	*±0.04*	0.32	*±0.03*	0.50	*±0.07*			
C18:1 n-7	4.47[bc]	*±0.10*	4.84[ab]	*±0.12*	4.09[cd]	*±0.23*	3.99[d]	*±0.10*		•••	
**Σ n-7**	**5.02[ab]**	***±0.14***	**5.44[b]**	***±0.15***	**4.41[a]**	***±0.25***	**4.49[a]**	***±0.14***		••	
C18:2 n-6	12.78[a]	*±0.29*	14.18[b]	*±0.30*	13.60[ab]	*±0.47*	14.18[b]	*±0.50*	***		
C20:3 n-6	0.34[a]	*±0.04*	0.56[b]	*±0.04*	0.40[b]	*±0.02*	0.42[b]	*±0.04*	***	•	*
C20:4 n-6	22.62[a]	*±0.26*	21.32[a]	*±0.47*	13.83[b]	*±0.35*	13.71[b]	*±0.24*		•••	
C22:4 n-6	0.65[a]	*±0.06*	0.80[a]	*±0.06*	0.08[b]	*±0.02*	0.09[b]	*±0.02*		•••	
C22:5 n-6	0.78[a]	*±0.08*	0.73[a]	*±0.05*	tr		tr			•••	
**Σ n-6**	**37.18[a]**	***±0.29***	**37.58[b]**	***±0.51***	**27.91[c]**	***±0.53***	**28.40[c]**	***±0.45***	*	•••	
C18:3 n-3	0.19	*±0.02*	0.28	*±0.03*	0.16	*±0.02*	0.20	*±0.03*	*		
C20:5 n-3	0.13[a]	*±0.02*	0.15[a]	*±0.01*	0.38[b]	*±0.04*	0.37[b]	*±0.03*		***	
C22:5 n-3	0.96[a]	*±0.10*	1.46[b]	*±0.11*	0.53[a]	*±0.05*	0.63[a]	*±0.08*	***	•••	
C22:6 n-3	10.10[a]	*±0.52*	10.57[a]	*±0.51*	18.12[b]	*±0.85*	19.95[c]	*±1.02*	*	***	
**Σ n-3**	**11.37[a]**	***±0.60***	**12.46[a]**	***±0.58***	**19.20[b]**	***±0.81***	**21.16[c]**	***±1.07***	*	***	

For legend, see page 1.

Supplementary material 2 - Table 5: Red blood cells fatty acids composition of total lipids

	VO	DL	VO+DHA	DL+DHA	ANOVA LQ	ANOVA S	ANOVA LQ*S
C14:0	0.87 *±0.16*	0.71 *±0.07*	0.58 *±0.08*	0.95 *±0.14*	·		
C16:0	22.38^{a} *±0.81*	22.58^{a} *±0.63*	27.64^{b} *±0.42*	26.31^{ab} *±0.54*		*	·
C18:0	15.98 *±0.51*	17.04 *±0.39*	16.81 *±0.42*	16.97 *±0.49*		·	
**Σ SFA**	**39.22** ***±1.11***	**40.33** ***±0.91***	**45.03** ***±0.58***	**44.23** ***±0.75***			
C16:1 n-9	1.35 *±0.84*	0.41 *±0.10*	0.25 *±0.04*	0.26 *±0.04*			
C18:1 n-9	15.15 *±0.75*	14.10 *±0.83*	12.92 *±0.46*	12.96 *±1.24*			
**Σ n-9**	**16.50** ***±0.95***	**14.51** ***±0.89***	**13.17** ***±0.46***	**13.23** ***±1.25***			
C16:1 n-7	0.73 *±0.06*	0.89 *±0.12*	0.51 *±0.05*	0.57 *±0.05*			
C18:1 n-7	2.64^{a} *±0.09*	2.86^{a} *±0.11*	2.29^{b} *±0.04*	2.43^{b} *±0.08*	·	•••	
**Σ n-7**	**3.37**^{a} ***±0.11***	**3.75**^{c} ***±0.17***	**2.79**^{b} ***±0.07***	**3.00**^{ab} ***±0.07***	**	•••	
C18:2 n-6	5.89^{a} *±0.21*	6.99^{b} *±0.46*	6.87^{bc} *±0.13*	7.53^{c} *±0.36*	***	***	
C20:2 n-6	0.58 *±0.08*	0.41 *±0.05*	0.59 *±0.06*	0.57 *±0.09*			
C20:3 n-6	0.54^{a} *±0.04*	0.60^{a} *±0.04*	0.96^{b} *±0.06*	0.99^{b} *±0.07*		***	
C20:4 n-6	25.57^{a} *±0.70*	24.38^{b} *±0.88*	17.69^{c} *±0.40*	17.82^{c} *±1.07*	·	•••	
C22:4 n-6	1.24^{a} *±0.08*	1.24^{a} *±0.09*	0.42^{b} *±0.05*	0.60^{b} *±0.11*		•••	
C22:5 n-6	0.59^{a} *±0.05*	0.52^{a} *±0.04*	0.10^{b} *±0.04*	0.23^{b} *±0.06*		•••	
**Σ n-6**	**34.39**^{a} ***±0.71***	**34.14**^{a} ***±0.71***	**26.63**^{b} ***±0.39***	**27.73**^{b} ***±0.86***		•••	
C18:3 n-3	0.40 *±0.07*	0.67 *±0.12*	0.20 *±0.05*	0.24 *±0.06*	*		
C20:5 n-3	0.44^{a} *±0.11*	0.56^{a} *±0.08*	1.87^{b} *±0.13*	1.58^{b} *±0.19*		***	
C22:5 n-3	1.49^{a} *±0.14*	1.71^{b} *±0.16*	2.34^{bc} *±0.11*	2.63^{c} *±0.30*	**	**	
C22:6 n-3	4.19^{a} *±0.16*	4.32^{a} *±0.28*	7.97^{b} *±0.29*	7.36^{b} *±0.72*		***	
**Σ n-3**	**6.52**^{a} ***±0.32***	**7.26**^{a} ***±0.38***	**12.38**^{b} ***±0.33***	**11.81**^{b} ***±1.01***		***	

For legend, see page 1.
