# Supplementary material for: Incorporation of Dairy Lipids in the Diet Increased Long-Chain Omega-3 Fatty Acids Status in Post-weaning Rats
Source: Front Nutr. 2018 May 23;5:42. doi: 10.3389/fnut.2018.00042 (PMC5974923; doi:10.3389/fnut.2018.00042)
Supplement: Supplementary file 3 [file Table_3.PDF]

# *Supplementary Material 3*

## Dairy lipids enriched diet increased Omega-3 status in post-weaning rats.

**Gaetan Drouin[1], Daniel Catheline[1], Anaëlle Sinquin[1], Charlotte Baudry[2], Pascale Le Ruyet[2], Vincent Rioux[1], Philippe Legrand[1*]**

*** Correspondence:** Corresponding Author: philippe.legrand@agrocampus-ouest.fr

**1 Supplementary material 3 - Figure 1: Principal component analysis of the heart**

**2 Supplementary material 3 - Figure 2: Redundancy analysis of the heart**

**3 Supplementary material 3 - Table 1: Pearson correlation coefficient of heart redundancy analysis**

**Supplementary material 3 - Figure 1: Principal component analysis of the heart**

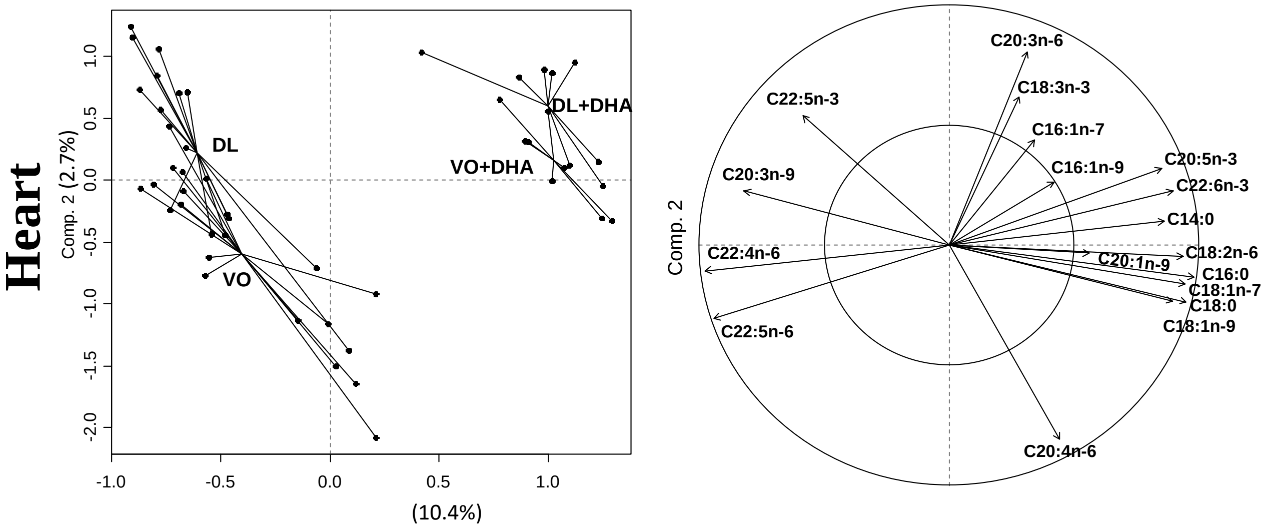


PCA individuals score plots **(A)** and PCA loadings plots **(B)** of heart (n=48) fatty acids compositions, of rats fed during 6 weeks with the four experimental diets. Percentages expressed the explained variance associated with each component.

**Supplementary material 3 - Figure 2: Redundancy analysis of the heart**

A

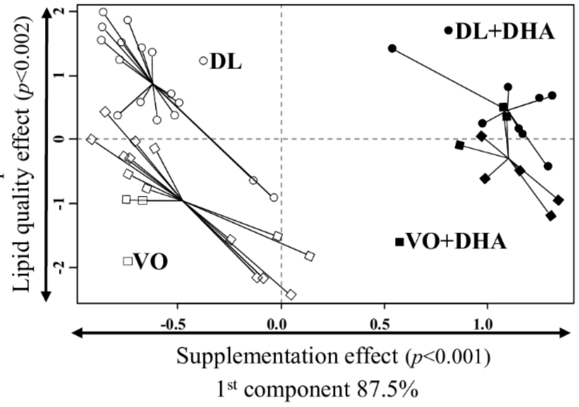


C

| RDA pairwise comparisons | | | |
|---|---|---|---|
| | **DL** | **DL+DHA** | **VO** |
| **DL+DHA** | 0.0012 | - | - |
| **VO** | 0.0012 | 0.0012 | - |
| **VO+DHA** | 0.0012 | 0.0230 | 0.0012 |

B

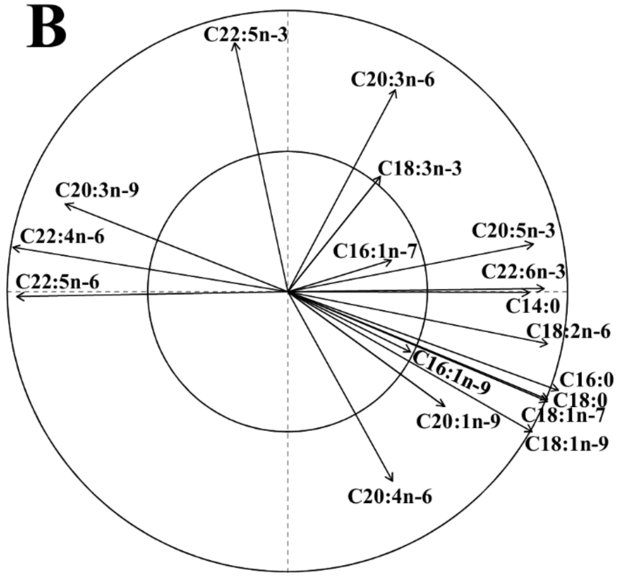


**Results:** In the heart, the DHA supplementation effect can be decomposed along the first component and the lipid quality effect along the second component **(A-B)**. To explain the differences in the FA profiles, the DHA supplementation effect influenced the variability (87.5% of constrained variance) more than the lipid quality effect (10.8%). Surprisingly, the proportions of n-3 DPA and C20:3n-6 are the only one that discriminated DL diets well while arachidonic acid (C20:4n-6) discriminated VO diets ($R^2>0.5$). The model expressed 58.6% of constrained variability and all groups were significantly discriminated ($p<0.01$) **(C)**.

**Legend:** Redundancy analysis was performed to assess differences in fatty acids metabolic profile in heart of rats fed the vegetable oils blend diet (VO) or the vegetable oil and dairy lipid blend diet (DL); not supplemented or supplemented with 0.5% DHA (VO+DHA and DL+DHA) for 6 weeks (n = 8/group for rats supplemented with DHA and n = 16 for rats not supplemented for 6 weeks). We considered as constraints the lipid quality effect, the DHA supplementation effect and the interaction between these two effects. **(A)** Scatter plots showed individual scores. **(B)**: Loadings plots showed correlation coefficient between fatty acids data and individual scores, a Pearson correlation coefficient procedure was performed to consider the significance of FA for each component (See Supplementary Material 3, Table 1). **(C)**: A permutation test adjusted with false discovery rate method was used to consider the significance of the lipid quality effect (LQ) (VO+VODHA *vs* DL+DLDHA) and the DHA supplementation effect (S) (VO+DL *vs* VODHA+DLDHA). This test was followed by pairwise comparisons using factor fitting to an ordination adjusted with false discovery rate method to assess the significance between the 4 groups.

**Supplementary material 3 - Table 1: Pearson correlation coefficient of heart redundancy analysis**

| RDA Pearson correlation coefficient and test between FA and individuals | | | | | | | |
|---|---|---|---|---|---|---|---|
| First component | | | | Second component | | | |
| **Fatty acids** | **Corr** | **P.value** | | **Fatty acids** | **Corr** | **P.value** | |
| C16 :0 | 0.9657 | 1.2639e-27 | *** | C22 :5 n-3 | 0.915000 | 3.2251e-11 | *** |
| C18 :2 n-6 | 0.9267 | 1.4619e-20 | *** | C20 :3 n-6 | 0.716800 | 9.0481e-08 | *** |
| C18 :1 n-7 | 0.9262 | 1.4619e-20 | *** | C18 :3 n-3 | 0.407300 | 1.2248e-02 | * |
| C18 :0 | 0.9244 | 2.1211e-20 | *** | C20 :3 n-9 | 0.310400 | 5.7171e-02 | . |
| C22 :6 n-3 | 0.9145 | 2.7529e-19 | *** | C20 :5 n-3 | 0.169200 | 3.4663e-01 | |
| C20 :5 n-3 | 0.8735 | 1.2473e-15 | *** | C22 :4 n-6 | 0.156700 | 3.6968e-01 | |
| C18 :1 n-9 | 0.8702 | 1.9226e-15 | *** | C16 :1 n-7 | 0.109900 | 5.4831e-01 | |
| C14 :0 | 0.8604 | 8.3356e-15 | *** | C22 :6 n-3 | 0.011130 | 9.7696e-01 | |
| C20 :1 n-9 | 0.5577 | 5.7256e-05 | *** | C14 :0 | -0.004282 | 9.7696e-01 | |
| C16 :1 n-9 | 0.4386 | 2.3411e-03 | ** | C22 :5 n-6 | -0.019210 | 9.7696e-01 | |
| C20 :3 n-6 | 0.3849 | 8.2918e-03 | ** | C18 :2 n-6 | -0.188100 | 3.0061e-01 | |
| C20 :4 n-6 | 0.3715 | 9.9915e-03 | ** | C16 :1 n-9 | -0.214100 | 2.3560e-01 | |
| C16 :1 n-7 | 0.3710 | 9.9915e-03 | ** | C16 :0 | -0.350400 | 2.9226e-02 | * |
| C18 :3 n-3 | 0.3295 | 2.2195e-02 | * | C18 :1 n-7 | -0.384400 | 1.5736e-02 | * |
| C22 :5 n-3 | -0.5347 | 1.2514e-04 | *** | C18 :0 | -0.389600 | 1.5736e-02 | * |
| C20 :3 n-9 | -0.7919 | 3.3408e-11 | *** | C20 :1 n-9 | -0.407100 | 1.2248e-02 | * |
| C22 :5 n-6 | -0.9643 | 2.0746e-27 | *** | C18 :1 n-9 | -0.497900 | 1.4254e-03 | ** |
| C22 :4 n-6 | -0.9795 | 2.1434e-32 | *** | C20 :4 n-6 | -0.673100 | 9.5813e-07 | *** |

Test of Pearson correlation coefficient between FA data and individual scores. This test was used to show FA explaining most of the variability corresponding to the component representing the LQ effect or S effect on the loadings plots.
