# Supplementary material for: Incorporation of Dairy Lipids in the Diet Increased Long-Chain Omega-3 Fatty Acids Status in Post-weaning Rats
Source: Front Nutr. 2018 May 23;5:42. doi: 10.3389/fnut.2018.00042 (PMC5974923; doi:10.3389/fnut.2018.00042)
Supplement: Supplementary file 4 [file Table_4.PDF]

Supplementary Material 4

Dairy lipids enriched diet increased Omega-3 status in post-weaning rats.

Gaetan Drouin[1], Daniel Catheline[1], Anaëlle Sinquin[1], Charlotte Baudry[2], Pascale Le Ruyet[2], Vincent Rioux[1], Philippe Legrand[1*]

*** Correspondence:** Corresponding Author: philippe.legrand@agrocampus-ouest.fr

Supplementary material 4 – Figure 1: Δ-6 desaturase activity and Fads2 mRNA relative expression

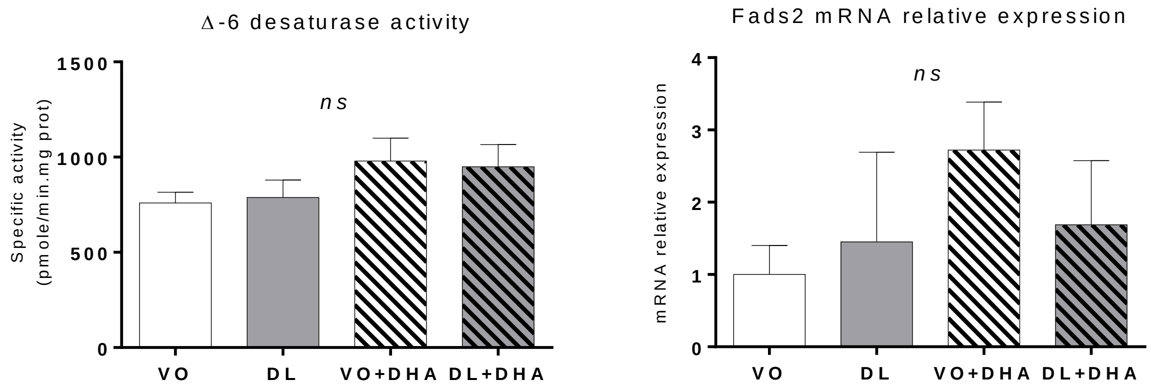

Values are represented as mean ± SEM (n = 8/group). A Kruskal-Wallis test followed by a post-hoc test of Dunn's were used to compare groups. Two different letters indicate significantly different values. *ns* = non significant. Fads2 : Fatty acid desaturase 2 gene.
